# Nutritional missed opportunity costs: wild house mice ( *Mus musculus* ) consistently consume less preferred food, with implications for control

**DOI:** 10.1002/ps.70229

**Published:** 2025-09-17

**Authors:** Finn C. G. Parker, Catherine J. Price, Jenna P. Bytheway, Peter B. Banks

**Affiliations:** ^1^ School of Life and Environmental Sciences, The University of Sydney Sydney Australia

**Keywords:** baiting, foraging, decision‐making, nutritional geometry, rodent, pest management

## Abstract

**BACKGROUND:**

Foraging animals must navigate trade‐offs among foods of varying quality and accessibility, with preferences generally predicted to be driven by energy maximisation. Limited access to preferred resources increases missed opportunity costs (MOCs), which should necessitate uptake of less‐preferred alternatives. How animals trade off access to preferred *versus* less‐preferred foods has important implications for wildlife management, especially where anthropogenic, natural, and management‐related food sources co‐occur. In cropping systems, mouse control relies on their uptake of unpalatable poisons coated on wheat grains, even when high‐quality alternatives like sown and spilled grain are available. We tested whether increasing the cost of accessing preferred wheat seeds would shift mice toward consuming a less‐preferred food, lentils.

**RESULTS:**

We conducted two experiments manipulating the costs of searching for wheat, predicting mice would compensate by eating more lentils. Unexpectedly, mice ate similar quantities of lentils across treatments, regardless of wheat accessibility. We suggest this occurred because lentils contain substantially more protein than wheat, leading to a nutritional MOC associated with wheat – that is, a fitness cost incurred from an imbalanced, wheat‐only diet.

**CONCLUSION:**

MOCs are rarely defined and are typically framed as time or energy trade‐offs, yet animals also incur fitness costs from nutritional imbalance. Bait substrates usually target pest preferences, but uptake may be limited when substrates match the macronutrient composition of background food (e.g., crops). We suggest that bait uptake could be improved by considering nutritional MOCs and selecting substrates that complement the macronutrient composition of background alternatives, thereby exploiting animals' nutritional requirements. © 2025 The Author(s). *Pest Management Science* published by John Wiley & Sons Ltd on behalf of Society of Chemical Industry.

## Graphical abstract

Wild house mice consistently consumed a less‐preferred, protein‐rich food despite access to a higher‐energy, preferred option. This suggests that fitness costs from imbalanced diets – nutritional missed opportunity costs – can override preference and energy maximisation, highlighting the potential to improve bait uptake by selecting bait substrates that exploit pests' nutritional requirements.

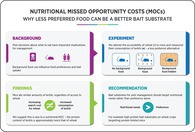

## 1 INTRODUCTION

Wild animals constantly make decisions consequential to their fitness, particularly regarding food.^1^ Foraging theory predicts that taking the highest quality foods in the safest areas will yield the greatest fitness benefits, but this situation is rare. High quality food is often not available,^2^ unsafe to access,^3^, ^4^ the cost of pursuing it (e.g., time or energy) is prohibitively high,^5^ or a combination of factors. However, animals cannot simply stop foraging,^6^ but instead must make trade‐offs such as safety against food quality,^4^ or food quality against accessibility.^7^

Resource switching (i.e., shifting from foraging for one food to another) is crucial for survival and fitness when foods fluctuate spatially and temporally in the environment. Although typically linked to changes in resource or prey density, the mechanism for switching is likely the increased foraging costs relative to alternatives.^8^ Animals should begin searching for alternative foods when the time or energy required to find a preferred option becomes too high.^5^, ^9^ Thus, the decision to switch is driven by missed opportunity costs (MOCs), which are the fitness gains lost by foregoing other foraging opportunities, which optimally foraging animals will minimise.^5^, ^10^ Limited access to preferred foods should prompt animals to search for lower quality alternatives, but the trade‐off between food quality (e.g., energy or calorie content) and accessibility remains poorly explored (but see Price and Banks^11^ and Norbury^12^).

Foraging animals will preference certain food types provided they are available,^13^ although the reasons for such preferences are not always well known. Classical foraging theory focuses on energy as the primary currency by which foraging trade‐offs are measured.^10^, ^14^ Thus, animals are often predicted to preferer resources that yield the highest net energy gain (energetic intake minus costs associated with searching, handling, etc.).^14^, ^15^ In this case, decreased accessibility to attractive options (i.e., more energy required to find them) should increase consumption of less preferred alternatives.^16^, ^17^ However, preference could also be driven by other factors, including risk,^18^ taste,^19^ toxins,^20^ or nutritional composition.^21^

How animals decide between foods underpins wildlife management, which depends on animals choosing to interact with, for example, baits or lures^22^, ^23^, ^24^ despite the presence of alternative foods, for example, anthropogenic food or crops or threatened prey.^24^, ^25^ Yet, despite its importance, the specific motivators for foraging decisions in this context are not well understood. Any attractive alternatives that are available will increase the MOCs of interacting with a management stimulus, resulting in poor uptake. This is why securing food stores^26^ and reducing access to food, water and shelter^27^ are considered important for managing pest animals, that is, to remove as many potentially better alternatives as possible.^28^ However, increasing the perceived costs of searching for preferred options should mean pests will be more likely to consume less preferred options.^29^

In Australia, house mice (*Mus musculus*) are a significant agricultural pest and are managed using poisons such as zinc phosphide‐coated cereal grains like wheat, which is considered mice's preferred food.^30^ Zinc phosphide is odorous,^31^ and strong behavioural aversions that mice form following exposure to sublethal doses indicate it is detectable.^32^ Mice are olfactory foragers that can exhibit neophobia towards novel food odours,^33^ and low bait uptake suggests they perceive zinc phosphide baits as unattractive. House mice live in agricultural crops year‐round,^34^ surviving off background food alternatives, especially spilled wheat from previous harvests.^35^ Huge influxes of food also become available when crops are sown,^36^ making less preferred foods (e.g., baits) both hard to find and less appealing when found. Baiting programmes thus rely on mice finding and consuming an unpalatable food item amidst a background of abundant, palatable alternatives. Given poison is already applied to what is thought the most attractive food option (wheat), reducing access to background food might improve bait uptake.

An emerging method to limit the accessibility of wheat to mice by increasing search costs is odour camouflage. Odour camouflage is a form of olfactory misinformation that disrupts the ability of olfactory foragers to find target foods.^37^ Odours that match a target food are dispersed throughout the environment, creating a uniform odour distribution that makes foods much more difficult to find without the patchiness in odour required to accurately locate foods.^38^ This method significantly reduces damage to wheat crops by wild mice,^39^ because when a food requires too much time or energy to find (i.e., the search costs become too high), foragers must look for alternative food sources. We predicted that odour camouflage of a preferred food may thus improve consumption of less preferred foods.

We conducted two experiments to test how the availability and accessibility of a highly palatable and preferred food (wheat) influences mouse consumption of a less‐preferred food option (lentils). We used lentils as a proxy for an unpalatable bait, because house mice almost completely avoid lentils when wheat is available in laboratory settings.^28^ Both grains have similar gross energy content,^28^ but higher fat and starch content, and lower fibre in wheat likely make its digestible energy greater for mice.^40^, ^41^, ^42^, ^43^, ^44^ Moreover laboratory animals also generally show a preference for fat.^44^ Thus, lentil consumption should be highest when wheat is unavailable or hard to find (MOCs are lowest).

In both experiments, we used the giving‐up density (GUD) framework^5^ to measure foraging responses to manipulated costs of searching for wheat. GUDs reflect patch‐quitting decisions driven by foraging cost, predation risk and MOCs,^5^ allowing us to observe how food consumption changes in response to search costs and MOCs. In experiment 1, we altered the MOCs associated with freely available lentils by varying access to wheat in three treatments: (i) wheat freely available in GUD trays; (ii) wheat removed entirely; and (iii) wheat available but treated with an odour camouflage (wheat germ oil solution) to increase olfactory search costs. In experiment 2, we had control and camouflage treatments where wheat and lentils were available in GUD trays. In camouflage treatments, some wheat trays had 10× and 50× odour camouflages. We compared lentil and wheat consumption between treatments in both experiments, and mouse foraging effort between treatments in experiment 1. We predicted that lentil consumption would be lowest when MOCs were high, that is, wheat was freely available and easily detectable (not camouflaged), and highest when MOCs were low, that is, mice perceived a lack of wheat (either due to camouflage or because food is not available). We also predicted that mice would exert the most effort searching for wheat when it was camouflaged but eat more lentils to compensate for extra foraging effort.

## 2 MATERIALS AND METHODS

**Figure 1:**
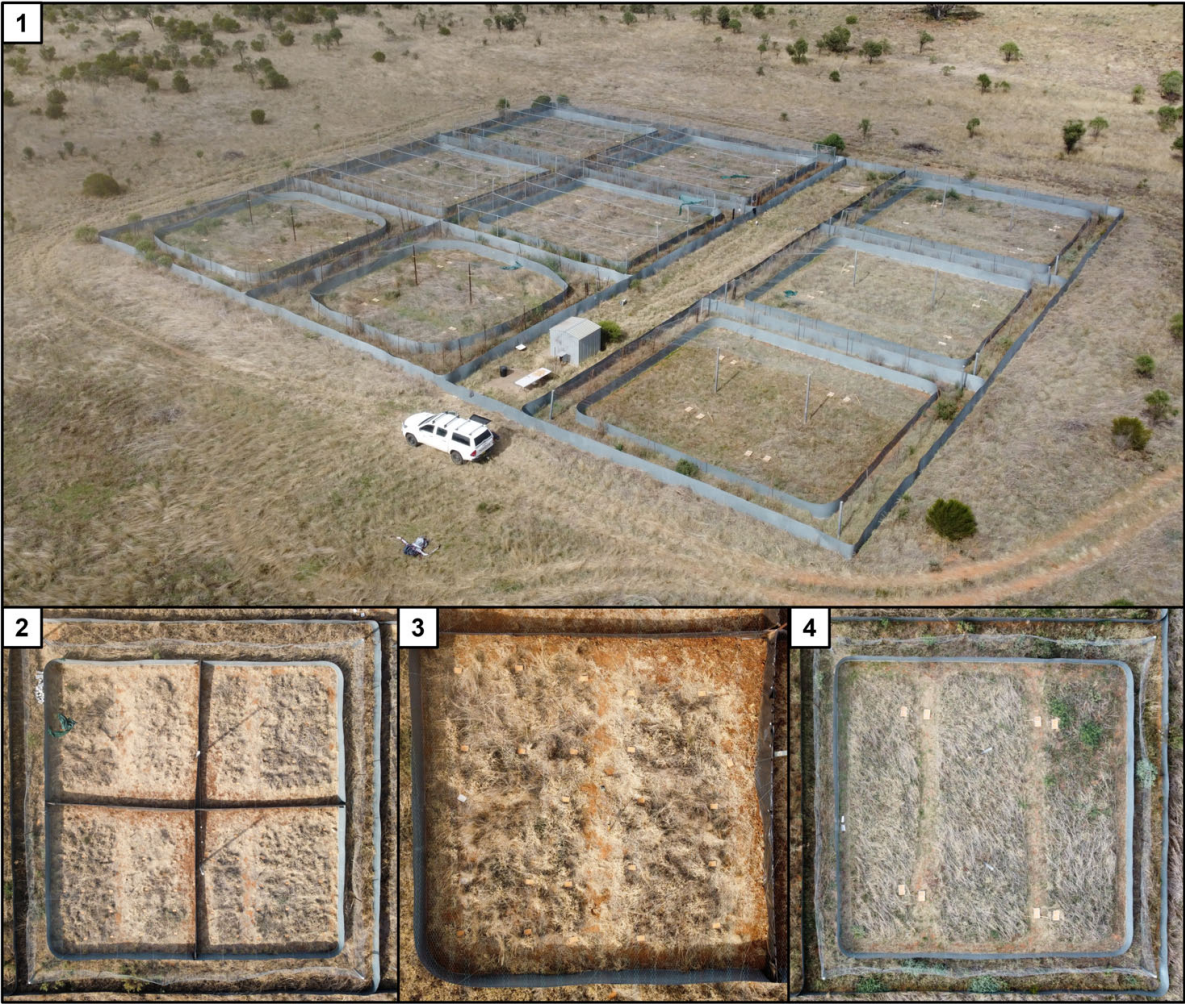
(1) Aerial view of the nine mouse‐ and predator‐proof enclosures used in this study, located in Walpeup, Victoria, Australia. (2) An enclosure divided into four sub‐enclosures (7.5 m × 7.5 m) for experiment 1. Fencing wire was strung up across the middle of the enclosure in both directions, fixed to the existing fence structure at the edges of the enclosure, and supported by a central pole fixed in the ground. Plastic sheeting was hung over the wire and buried into trenches. (3) A close‐up view of a sub‐enclosure used for experiment 1. Twenty‐five patches (food trays) have been arranged in a 5 × 5 grid, approximately 1.2 m away from the edge of the enclosure and from the nearest other patches. (4) Top‐down aerial view of one of the enclosures (15 m × 15 m) used to conduct experiment 2. Note that this image shows food trays setup for a different study but accurately depict vegetation strips and trays match patch locations (two patches in opposite corners).

Both experiments were conducted using nine 225 m^2^ outdoor field enclosures in Walpeup, Victoria, Australia (−35.138336, 142.023412). Enclosures comprised a perimeter fence measuring 15 m × 15 m with an additional outer fence providing a 1‐m buffer around each enclosure (Fig. 1(1),(2)). Enclosures contained grass and vegetation typical of the surrounding area, and were mouse‐ and predator‐proof, with two layers of smooth, galvanised steel approximately 1.5 m high and buried 50 cm underground to prevent mice digging under or climbing over it (Fig. 1(1)) for additional details, see Barker *et al*.^45^

### 2.1 Experiment 1

#### 2.1.1 Enclosures

Experiment 1 was conducted in April–May 2024, using three of nine enclosures subdivided into four equally sized smaller enclosures (7.5 m × 7.5 m) (Fig. 1(2)). This was achieved by digging two, 30 cm‐deep, intersecting trenches across the enclosure, hanging polyethylene sheets from fencing wire strung above the trenches, and burying the sheets into the trenches. Vegetation within 50 cm of the edge was removed around the entire inner perimeter of each enclosure to discourage mice from foraging close to fences.

#### 2.1.2 Trapping and setup

Mice that were already present in enclosures were removed using Elliott traps, with trapping continuing until enclosures were deemed mouse‐free (three consecutive nights with no captures). Captured mice were transferred to separate holding enclosures. Once experimental enclosures were mouse‐free, mice were trapped out from holding enclosures, weighed, sexed, uniquely marked, and one mouse was allocated to each of the 12 sub‐enclosures. Mice were not returned to the same enclosure they were trapped from, to prevent knowledge about enclosures influencing foraging decisions.^46^ Pregnant females and subadults (< 11 g) were excluded from the study.

Each sub‐enclosure had 25 ‘patches’ deployed in a 5 m × 5 m grid, with each patch at least 50 cm away from the edge of the enclosure and approximately 1 m apart from each other (Fig. 1(3)). Each patch consisted of a plastic container (172 mm × 120 mm × 39 mm) containing 500 mL of sand collected from a nearby paddock (< 3 km away) that had been sieved to remove debris. A single Petri dish containing 20 red lentils was placed in the centre of the enclosure, with two cameras staked into the ground on either side to record mouse visitations to the lentil dish (BolyGuard SG2060‐X, settings: 30 s video, 0 s interval between triggers, high sensitivity, 1280 × 720 video resolution). Cameras were set to record visits between 5 p.m. and 7 a.m. (14 h). A visit was classified as whenever a mouse entered either of the two cameras' field of vision (approximately 40 cm × 40 cm) > 2 min after the last visit. Mice were given two nights to acclimatise to the experimental setup and learn to forage in patches for wheat and dishes for lentils before the treatments were applied. During this period, five of the 25 patches (one per row) were systematically selected to contain wheat (20 seeds), which was mixed through the sand. Remaining wheat seeds and lentils were counted each morning and replenished.

#### 2.1.3 Treatments

Following acclimatisation, each sub‐enclosure was randomly allocated to one of three treatments: wheat, no wheat, and camouflage. The ‘wheat’ treatment comprised 5/25 patches containing 20 wheat seeds. The location of the patches containing wheat seeds differed to the acclimatisation period to prevent spatial associations developing. In the ‘no‐wheat’ treatment, none of the 25 patches contained wheat, leaving lentils as the only food source, besides any background food present in the enclosure. The ‘camouflage’ treatment was similar to the ‘wheat’ treatment (5/25 patches containing wheat seeds in new locations) but with the surface of the sand in all 25 patches additionally sprayed with a wheat germ oil solution, that is, an odour camouflage, see Parker *et al*.^39^ Camouflaging trays with wheat odour should prevent mice from accurately locating the trays containing wheat.^38^, ^39^ Wheat germ oil quantities were measured out to simulate the smell of approximately 50 times the number of seeds in each tray (1000 seeds). Each 1 mL of wheat germ oil (organic cold pressed; Leonardi Laboratories, Sydney, NSW, Australia) requires approximately 5000 seeds^47^ for more detailed calculations, see Parker *et al*.^39^ Thus, 0.2 mL of wheat germ oil per tray (5 mL per enclosure) was mixed with water in a roughly 10:1 ratio and sprayed over patches on both nights that the experiment was running. In ‘wheat’ and ‘camouflage’ treatments, the location of patches containing wheat were moved systematically (i.e., two places across in each row) between night 1 and night 2.

#### 2.1.4 Data collection

Each morning, the remaining lentils in the dish and the number of wheat seeds remaining (i.e., the GUD) were counted. Wheat GUDs were averaged across each of the five patches to get a single value for each enclosure. The number of patches visited and dug up were also recorded and used as a metric for foraging effort. A patch was classified as visited if there was any evidence that a mouse had entered the tray, for example footprints or small depressions in the sand where a mouse had put its nose (Supporting Information, Fig. S1). A patch was classified as having been dug up if there were any clear holes in the sand's surface, and in some cases removal of sand from the tray entirely (Fig. S1). Lentils and wheat were replenished and the surface of the sand in trays smoothed between night 1 and night 2.

In total, each mouse was in a sub‐enclosure for four consecutive nights (i.e., one trial, which comprised two acclimatisation nights followed immediately by two treatment nights). After the second treatment night, mice were trapped out of enclosures and replaced with new mice to begin a new trial. Sand trays were emptied, cleaned and replaced with new sand to ensure no carryover of mouse odour from previous trials. Three trials were run in each enclosure, with a new treatment being allocated to an enclosure each time. Two trials were excluded because there was no evidence of mouse activity on any night. If there was evidence of more than one mouse in an enclosure, the minimum number known alive (MNKA) was recorded, and included in our analysis if MNKA < 3 (all trials except one). One trial where MNKA = 3 was excluded. Our final sample size was 11 no‐wheat trials, ten wheat trials, and nine camouflage trials.

#### 2.1.5 Statistical analysis

All statistical analysis was conducted in R (version 4.4.1;^48^), and data were visualised using the ‘ggplot2’ package.^49^ All final models were selected using Akaike's information criterion (AIC),^50^ with the best‐fitting models chosen based on the lowest AIC value. *Post hoc* comparisons of significant interactions were conducted using estimated marginal means (EMMs) with the ‘emmeans’ package, adjusted for multiple comparisons.^51^ All model assumptions were validated using residual diagnostics with the ‘DHARMa’ package.^52^

The number of lentils remaining each morning was right skewed (i.e., many lower values), overdispersed (variance greater than mean), and included a high proportion of zeroes (29/60). Because the response variable was a bounded count (i.e., lentils removed out of a fixed total), we modelled the data using a generalised linear mixed model (GLMM) with a beta‐binomial distribution and logit link using the ‘glmmTMB’ package.^53^ This approach accounts for overdispersion in binomial data while allowing for random effects. The final model included fixed effects for treatment, night, trial, and MNKA, with enclosure included as a random effect to account for repeated measures and variation among enclosures.

The number of visits to lentil dishes were analysed using a linear mixed effects model (LMM) in the ‘lme4’ package,^54^ with enclosure included as a random effect. Fixed effects were tested separately for treatment, night, MNKA, and trial, with significance assessed using Type II Wald chi‐square (*χ* ^2^) tests using the Anova function in the ‘car’ package.^55^ To examine how visits to lentil dishes varied between treatments early in the night, we divided the night into four equal quarters (17:00 h–07:00 h, 3.5‐h increments) and calculated the mean number of visits per quarter of the night for each treatment. We focused on the first quarter because early visits reflect how willing mice were to forage for lentils initially across treatments. Visits to treatments in the first quarter of the night were compared using a negative binomial regression with treatment, night, trial, and MNKA as fixed effects and enclosure as a random effect.

The number of patches visited and dug up were used to index the foraging effort mice allocated to finding wheat. In some enclosures, many patches were visited, leading to a heavily left‐skewed distribution of the ‘patches visited’ variable (i.e., many high values). Additionally, only 25 patches were present, so the variable was bounded at 25 patches visited. The number of patches visited (out of 25) and the number of patches dug up were modelled separately. We used a beta‐binomial regression with a logit link to model patches visited, accounting for the bounded nature of the data and overdispersion. The model was fitted using the ‘glmmTMB’ package.^53^ Treatment, night, MNKA, and trial were initially included as fixed effects, and interactions between main effects were tested. The final model was selected using AIC, with treatment and MNKA retained as fixed effects in both final models. Enclosure was included as a random effect to account for repeated measures.

We used LMMs to test how wheat GUDs differed as a function of treatment, trial, MNKA, and night, and for any interactions between main effects. Enclosure was included as a random effect to account for repeated measures. The final model included treatment, trial, and MNKA, as well as treatment × MNKA and treatment × night interactions.

### 2.2 Experiment 2

#### 2.2.1 Enclosures and trapping

In experiment 2, we used the GUD approach in larger enclosures containing multiple mice, with lentils offered in a foraging matrix (i.e., sand trays) rather than freely available. Six whole enclosures were used in April–May 2023. Within each enclosure, vegetation around the perimeter was mowed to create a 50 cm buffer zone from the enclosure edge, to discourage mice from foraging in or inhabiting areas close to the fence. Two parallel strips the length of the enclosure (~1 m wide) mowed to create open areas that represented uniform predation risk, rather than heterogenous ground cover. These strips were approximately 6 m apart and 3 m from the side perimeter (Fig. 1(4)). Mice were already present in enclosures, so we continually trapped and marked mice using live trapping (Elliott traps), before beginning to remove trapped mice until all enclosures had an MNKA of approximately 10–12. We also selectively removed mice in order to maintain an even sex ratio, minimising potential for confounding social and sex‐related factors.^56^

#### 2.2.2 Setup and data collection

Food trays (foil BBQ trays; 32.5 cm × 26.3 cm × 6.7 cm) contained 2.5 L of sifted sand as the food matrix. Each enclosure contained two ‘patches’ in opposite corners of the enclosure at the end of the mowed strips (Fig. 1(4)). A patch consisted of four GUD trays arranged in a 2 × 2 grid immediately beside one another (Fig. S2). Half of the enclosures were initially randomly allocated as control enclosures (*N* = 3) while the other half were allocated as camouflage enclosures (*N* = 3). In control enclosures, three of the four trays in each patch were randomly selected to contain 30 wheat seeds, while the remaining tray contained 30 whole green lentils. This amount of wheat and lentils provided adequate food for mice but ensured mice were motivated to forage. In camouflage enclosures, the setup was identical except that one of the wheat trays in each patch was randomly allocated as a 10× camouflage treated tray, and another as a 50× camouflage treated tray. In these trays, the surface of the sand was sprayed with a wheat germ oil and water solution to increase the search costs of finding wheat using olfaction. Wheat germ oil quantities equated to 10 and 50 times the number of seeds in a tray (300 and 1500 seeds, respectively) and were calculated as in experiment 1. Six trays (two per enclosure) equated to 0.36 mL for the 10× treatment and 1.8 mL for the 50× treatment, both mixed into 100 mL of water. Trays were sprayed before being randomly allocated to a position within each 2 × 2 patch.

Prior to treatments being applied, all enclosures underwent a one‐night acclimatisation period, so that mice could adjust to the experimental setup. During this period, all enclosures were set up in the same way as control enclosures. One night acclimatisation was deemed sufficient, as mice in enclosures had already learned to forage in GUD trays from a previous experiment conducted with entirely different foods. After one night, treatments were applied, and the number of seeds remaining in each tray (i.e., the GUD) were counted each morning for two nights. Treatments were then swapped, with control enclosures becoming camouflage enclosures and *vice versa*. New trays and new sand were used for each treatment in all enclosures. GUDs were counted each morning for a further two nights.

#### 2.2.3 Statistical analysis

We fitted a LMM with the ‘lme4’ package^54^ to test how lentil and wheat GUDs varied as a function of our fixed effects: enclosure treatment (control *versus* treatment), contents (wheat *versus* lentils), and tray treatment (wheat only, wheat with 10× camouflage, wheat with 50× camouflage). Enclosure was included as a random effect to account for variability across enclosures. Models were fitted using restricted maximum likelihood (REML) and evaluated using *t*‐tests for fixed effects. Model assumptions were validated using residual diagnostics with the ‘DHARMa’ package.

## 3 RESULTS

### 3.1 Experiment 1

#### 3.1.1 Lentils remaining

**Figure 2:**
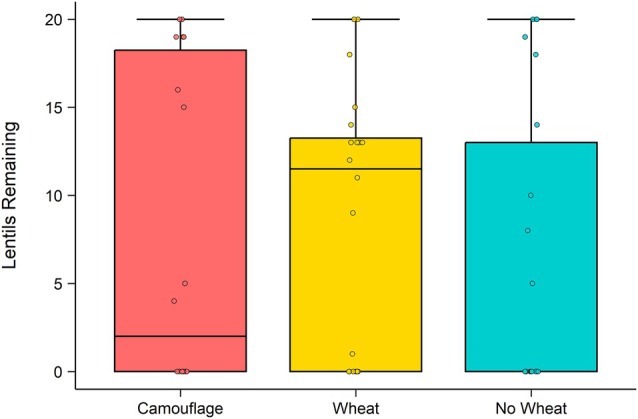
Number of lentils remaining per night across the three experimental treatments: camouflage (red; *N* = 9), wheat (yellow; *N* = 10), and no wheat (blue; *N* = 11) over both nights of the experiment. Boxes show the interquartile range (IQR) (box limits), median line, 1.5 times the IQR (box whiskers), outliers (points). Jittered points are offset slightly to avoid overlap and aid visualisation of raw data.

Contrary to predictions, mice removed similar numbers of lentils across all treatments (Fig. 2). That is, there was no significant effect of treatment on the proportion of lentils consumed (*χ* ^2^(2) = 1.36, *P* = 0.5). However, lentils were 5.6 times more likely to be removed when two mice were present in the enclosure (odds ratio (OR) = 5.6, 95% confidence interval (CI) [1.36, 22.91], *P* = 0.017). There was also a significant effect of trial on the odds of lentils being taken (*χ* ^2^(2) = 17.5, *P* < 0.0001), with lentils 23.4 times more likely to be removed in trial 2 (OR = 23.4, 95% CI [5.02, 109.2], *P* < 0.001) and 8.7 times more likely in trial 3 (OR = 8.7, 95% CI [2.37, 31.8], *P* = 0.001) compared to trial 1.

#### 3.1.2 Visits to lentil dishes

Over the whole night, mice made more visits to lentil dishes in no‐wheat enclosures (4.6 more visits per night) compared to camouflage enclosures (*t*(47.63) = 2.18, *P* = 0.034), although there was no overall effect of treatment (*χ* ^2^(2) = 5.38, *P* = 0.068). Night also had a significant effect on visits (*χ* ^2^(1) = 4.10, *P* = 0.040), with 3.2 fewer visits on night 2 compared to night 1 (*t*(39.15) = −2.02, *P* = 0.047).

**Figure 3:**
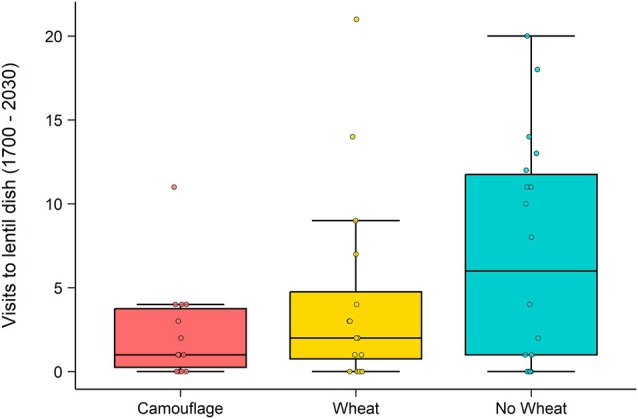
Average number of visits to lentil dishes per quarter of the night across three treatments: camouflage (red; *N* = 9), wheat (yellow; *N* = 10), and no wheat (blue; *N* = 11). Error bars represent ±1 standard error of the mean. Quarters of the night are divided into 3.5‐h increments, which equates to one‐quarter of the total time that cameras were on and recording (17:00 h–07:00 h). Individual data points are jittered to display within‐group variation and reduce overlap.

In the first 3.5 h of the night (17:00 h–20:30 h), treatment did affect visits to lentil dishes (*χ* ^2^(2) = 6.8183, *P* = 0.033), with 3.3 times as many visits to lentil dishes in no‐wheat treatments than camouflage treatments (incidence rate ratio [IRR] = 3.25, 95% CI [1.33, 7.98], *P* = 0.01) (Fig. 3). There was no significant difference between wheat and camouflage treatments (*P* = 0.24). Night also varied in visits in the first 3.5 h (*χ* ^2^(1) = 6.75, *P* = 0.009), with 60% fewer visits on night 2 compared to night 1 (IRR = 0.4, *Z* = −2.6, *P* = 0.009).

#### 3.1.3 Wheat GUDs

Treatment also affected wheat GUDs (*χ* ^2^(1) = 5.18, *P* = 0.023), but there were significant treatment × MNKA (*χ* ^2^(1) = 17.07, *P* < 0.001), and treatment × night (*χ* ^2^(1) = 6.84, *P* = 0.009) interactions. *Post hoc* comparisons showed that when one mouse was present (MNKA = 1), GUDs were higher in camouflage treatments (*P* = 0.0013), but when two mice were present (MNKA = 2), GUDs were higher in wheat treatments (*P* = 0.001). On night 1, there was no significant difference in wheat GUDs between treatments (*P* = 0.57), but on night 2, GUDs were lower in camouflage treatments than wheat treatments (*P* = 0.018). Overall, wheat GUDs were significantly lower when two mice were present (*χ* ^2^(1) = 51.55, *P* < 0.001). Additionally, wheat GUD significantly decreased over successive trials (*χ* ^2^(2) = 89.49, *P* < 0.001), with an average of 4.5 fewer wheat seeds in trial 2 (*t*(22.8) = −5.43, *P* < 0.001) and 9.1 fewer in trial 3 (*t*(26.12) = −9.42, *P* < 0.001) compared to trial 1.

#### 3.1.4 Foraging effort (wheat patches visited and dug up)

**Figure 4:**
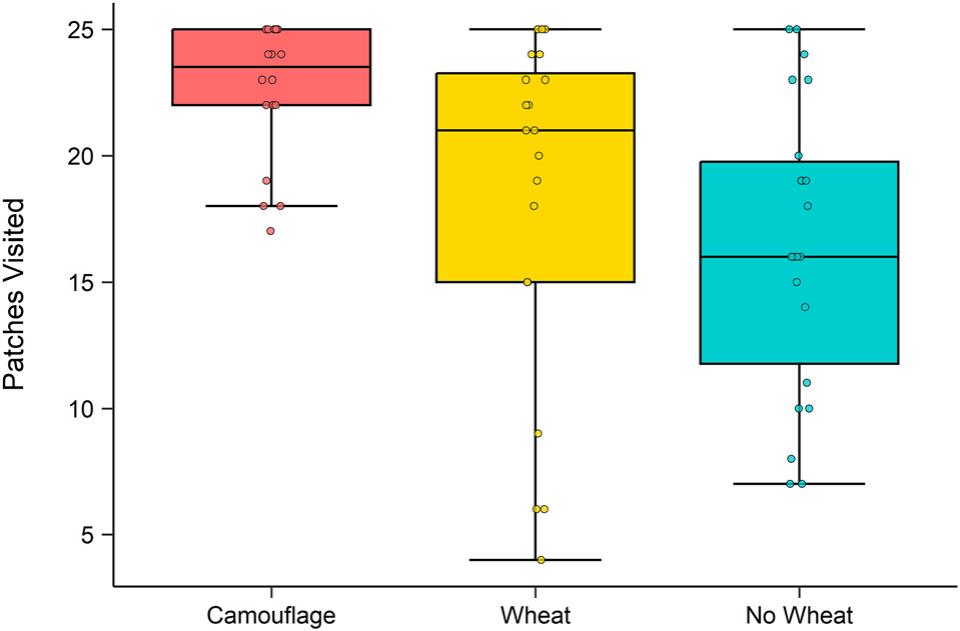
The number of patches visited each night across all enclosures of each of the three experimental treatments: camouflage (red; *N* = 9), wheat (yellow; *N* = 10), and no wheat (blue; *N* = 11). Boxes show the interquartile range (IQR) (box limits), median line, 1.5 times the IQR (box whiskers), outliers (points). Note that statistics in the text are reporting ‘patches unvisited’ (i.e., 25 – ‘patches visited’). Individual data points are jittered to display within‐group variation and reduce overlap.

Treatment affected both the number of wheat patches visited (*χ* ^2^(2) = 12.4, *P* = 0.002) and the number of wheat patches dug up (*χ* ^2^(2) = 24.7, *P* < 0.001) as measures of foraging effort (Fig. 4). Mouse foraging effort was highest in camouflage treatments, with mice visiting the most patches (22.1) and digging up the most patches (4.8) on average. Compared to camouflage treatments, the odds of a patch being visited were 73% lower in no‐wheat treatments (OR = 0.27, 95% CI [0.16, 0.53], *P* < 0.001) and 53% lower in wheat treatments (OR = 0.47, 95% CI [0.25–0.84], *P* = 0.066), although the latter difference was not statistically significant. Mice also dug up significantly fewer patches in both wheat and no‐wheat treatments compared to camouflage: the odds of digging up a patch were 48% lower in wheat treatments (OR = 0.52, 95% CI [0.28, 0.99], *P* = 0.045) and 84% lower in no‐wheat treatments (OR = 0.16, 95% CI [0.08–0.32], *P* < 0.001).

Foraging effort also increased when two mice were present (*χ* ^2^(1) = 4.0, *P* = 0.047 for patches visited; *χ* ^2^(1) = 11.8, *P* < 0.001 for patches dug up). When two mice were present, the odds of visiting a patch were 1.8 times higher (OR = 1.79, 95% CI [1.01, 3.13], *P* = 0.045), and the odds of a patch being dug up were 2.7 times higher (OR = 2.70, 95% CI [1.56–4.68], *P* < 0.001).

### 3.2 Experiment 2

**Figure 5:**
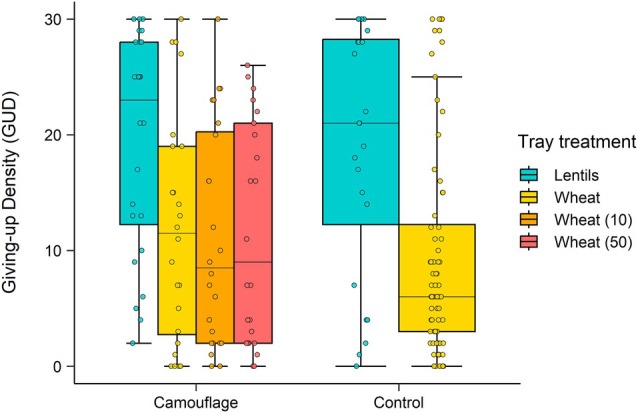
The giving‐up densities (GUDs) (i.e., the number of lentils remaining each night) in lentil trays (*N* = 24; 30 lentils per tray) and wheat (*N* = 72; 30 wheat seeds per tray) over two nights in camouflage and control enclosures. Camouflage enclosures had three treatments applied to wheat trays: wheat seeds only (*N* = 24), wheat seeds and 10× odour camouflage (*N* = 24), wheat seeds and 50× odour camouflage (*N* = 24). Boxes show the interquartile range (IQR) (box limits), median line, 1.5 times the IQR (box whiskers), outliers (points). Individual data points are jittered to display within‐group variation and reduce overlap.

Lentils were consumed in all treatments and odour treatments at wheat trays did not influence the number of lentils eaten. Overall, lentil GUDs (i.e., the number of lentils remaining each morning) tended to be lower in odour treatment enclosures compared to controls but not significantly (*t* = −1.8, df = 184, *P* = 0.08) (Fig. 5). There was a significant effect of tray contents on GUDs (*χ* ^2^(1) = 75.32, *P* < 0.001), with mice eating an average of 8.7 more wheat seeds than lentils (*t* = −8.68, df = 184, *P* < 0.0001), reducing GUDs by 43%. There was no significant difference in GUDs between any of the wheat trays, regardless of odour camouflage treatment, and mice left on average approximately ten wheat seeds per tray across both treatments. The random effect for enclosure accounted for variability between enclosures but did not strongly influence the fixed effects estimates. Residual diagnostics indicated that model assumptions were met.

## 4 DISCUSSION

In both experiments, we found that mice consumed the unpreferred lentils, regardless of treatment and when there was abundant alternative food available. Manipulating access to wheat (the preferred, attractive food) did not alter consumption of, or overall visitations to, lentil dishes, neither by camouflaging the wheat with odour, nor by removing the wheat entirely. In experiment 1, there was sufficient wheat available in wheat treatments for mice to fulfil their nightly energetic requirements,^57^ yet mice still consumed lentils similarly to when no wheat was available (approximately 17 lentils per night in both wheat and no‐wheat treatments, assuming all removed lentils were consumed). Our camouflage treatment did increase the foraging effort that mice allocated to searching for wheat (and thus, increased MOCs), as mice dug up more patches in camouflage than in wheat treatments. Yet, mice ate similar numbers of lentils to when wheat was available and uncamouflaged (~14 lentils), that is, regardless of MOCs. Clearly lentils are not as unpalatable as previously thought from laboratory trials. Instead, we suggest that mice consume lentils as well as wheat, which differ in nutrient composition, to balance macronutrient intake. Thus, we suggest consideration of food preference must include nutritional MOCs, as we found that mice's background preference for wheat was outweighed by their need to balance nutrient intake with easy‐to‐find lentils.

We predicted that increasing the costs of searching for wheat would lead to greater lentil consumption (i.e., due to low MOCs associated with lentils). Mice visited and dug up the most wheat patches in experiment 1 when wheat was camouflaged, and wheat could not be accessed in no‐wheat treatments, confirming that our treatments increased the costs of searching for wheat, yet lentil consumption was unaffected. In experiment 2, mice also consistently ate lentils, despite having to search for them in sand while wheat was available in adjacent trays. Previous laboratory trials show mice avoid lentils when wheat is available,^28^ likely due to lentils' lower digestible energy and lower fat content.^40^, ^41^, ^42^, ^43^, ^44^ Yet, if mice perceived MOCs purely in terms of energy, increased search effort for wheat should have led to greater lentil consumption. However, despite changes in energetic MOCs, lentil consumption remained consistent across treatments, which suggests lentil consumption was influenced by a factor other than energy maximisation. Optimal foraging models assume animals maximise energy intake while minimising energetic expenditure.^10^, ^14^ However, the nutritional geometry framework suggests that many animals regulate their nutrient intake to meet a specific nutritional target, an optimal ratio of nutrients at which fitness is maximised.^58^ Deviating from this target incurs a fitness cost,^59^ leading animals to adjust their feeding strategies to stay as close to this target as possible. Lentils contain roughly twice as much protein as wheat,^40^, ^41^, ^42^, ^43^ and wild house mice are generally protein‐limited in the wild.^60^ We suggest that, despite an overall preference for wheat, mice always consumed lentils because the fitness costs incurred from an imbalanced wheat‐only diet outweighed the MOCs of consuming a lower‐energy food – a nutritional MOC.

Exploiting food preferences is the basis for management techniques such as baiting, because they rely on animals accepting a food reward, often within a background of alternatives.^1^ Managers typically use no choice or limited choice preference trials of captive animals to select ‘preferred’ bait substrates,^61^ assuming they will be the most effective. In broadacre wheat cropping systems, mice are commonly baited using poisons coated onto cereal grains, including wheat.^30^, ^32^ However, wheat crops themselves provide a background of alternative food sources, including newly sown seeds and residual grain from previous harvests, which reduce the chances of finding baits and increase the MOCs of consuming them. Moreover, toxic grains have novel odours,^31^ which mice can display neophobia towards.^33^ Consequently, in this scenario, baits must be chosen in the presence of nutritionally similar (or equivalent) grains without unfamiliar odours. Our findings suggest that less preferred foods that meet specific nutritional requirements better than generally more attractive foods may represent a better bait option because they act on an animal's perception of its nutritional MOCs.

Considering the nutritional requirements of pests when selecting bait substrates therefore has the potential to improve the efficacy of baiting programmes. Captive mice naturally regulate their macronutrient intake,^62^ but in environments dominated by cereal grains, protein may be a limiting resource.^35^ Lentils contain > 20% protein,^40^ compared to approximately 12% in wheat.^42^ House mouse growth requires 12–14% protein, while reproduction requires 17–19% (as cited in Murphy^63^), implying a wheat‐only diet is barely sufficient for long‐term survival and insufficient for reproduction. Thus, bait substrates considered unpreferred but high in protein relative to background cereal grain alternatives may be a better option as they act on an animal's nutritional MOC, even if toxins are detectable on baits. Future research on baiting programmes should, therefore, consider nutritional MOCs when selecting potential bait substrates.

The unexpected consumption of lentils may be because mice simply do not prefer wheat over lentils as strongly as assumed. In experiment 1, mice in wheat treatments ate an average of 2.8 wheat seeds per tray across 5 trays, equalling 14 seeds per enclosure per night, which is roughly equivalent to 0.56 g wheat. Wild house mice eat up to and beyond 3 g of food per night, which equates to about 75 grains (0.04 g per seed).^57^ No mice came close to eating that many wheat seeds, despite them being available and uncamouflaged, which is surprising given their strong reported preference for wheat. However, visits to lentil dishes in no‐wheat treatments were more than three times higher in the first 3.5 h of the night than in camouflage treatments where wheat was available. Mice had to search and dig for wheat in camouflage enclosures yet initially appeared to choose working for wheat rather than taking lentils. If mice did not have a strong preference for wheat, then freely available lentils should have been sought first. That mice would work for wheat before taking lentils suggests that mice do prefer wheat in the absence of external influences such as food accessibility.

We included odour camouflage treatments to test whether increasing the search costs and MOCs associated with wheat would lead to greater lentil consumption. Odour camouflage has been used successfully in the field to decrease the foraging success of mice searching for wheat seeds,^39^ and is known to increase search costs of target foods in other contexts.^11^, ^38^, ^64^ In experiment 1, wheat GUDs were not lower in wheat treatments, suggesting that camouflage did not substantially reduce the ability of mice to find wheat, and, in fact may have motivated them to search for it in some circumstances. This is potentially due to the acclimatisation period, when mice learned that food was available in trays. It is common practice in GUD experiments to have an acclimatisation period to stabilise GUDs and allow foragers to learn that trays contain food.^65^ By the time camouflage was introduced, mice had already associated trays with food, and a strong food cue (wheat germ oil) may have further reinforced their motivation to continue searching, even if initially unsuccessful.^64^, ^66^

Although odour camouflage did not reduce wheat consumption, it did increase search effort, as evidenced by higher patch visitation and digging rates in camouflage enclosures. When a preferred resource becomes harder to find, optimal foraging theory predicts that animals will either switch to an alternative resource or intensify their search effort, for example, winter foraging behaviour.^67^ Here, rather than shifting entirely to lentils, mice increased their search effort for wheat in camouflage treatments, visiting more patches than in wheat treatments, despite wheat being equally available in both. One possible explanation for unchanged lentil consumption is that mice simply ate more lentils as the costs of searching for wheat increased over time. However, if increasing search costs alone drove diet shifts, we would expect higher lentil consumption in camouflage enclosures, where foraging was more energetically costly. This was not observed, reinforcing that mice did not consume lentils simply due to increasing wheat search costs.

## 5 CONCLUSION

That animals regulate their nutrient intake to meet nutritional targets^58^, ^59^, ^68^ is somewhat underappreciated in a wildlife management context. Food preference has traditionally been prioritised when selecting baits and lures for pest animals, but preference trials are often conducted in settings that fail to capture the environmental complexity and competing motivations that influence foraging decisions in the wild.^28^, ^61^, ^69^ Our findings highlight that animals may accept less preferred foods in order to meet macronutrient requirements, suggesting that bait uptake could be improved by using baits that complement the macronutrient composition of background foods (and thus, exploit nutritional MOCs).

In cropping systems, targeting mice using standard wheat bait substrates in wheat crops may lack efficacy given that pest nutritional requirements are already being met. Selecting bait substrates that complement the crop (e.g., protein‐rich baits in carbohydrate‐rich crops) could exploit animals' motivations to not miss out on essential macronutrients (i.e., incur a nutritional MOC). Alternatively, using a mixed‐substrate approach (e.g., wheat and lentils) could offer a more practical solution than tailoring bait types to each individual crop. Future research should compare uptake of bait substrates with different macronutrient compositions against various background food (crop) conditions, as well as mixed‐substrate approaches. Subsequent findings could potentially support the development of better baiting strategies. Moreover, targeting nutritional MOCs to improve bait uptake is likely applicable across a range of agricultural landscapes worldwide (e.g., maize, rice), where integrating nutritional ecology into baiting strategies could enhance pest management outcomes.

## CONFLICT OF INTEREST

The authors declare no conflict of interest.

## ANIMAL ETHICS

All research was approved by the University of Sydney's Animal Ethics Committee (protocol numbers 2021/1988 and 2024/2427).

## Supporting information


**Figure S1.** Plastic containers filled with sand that represented wheat ‘patches’ used in experiment 1. The left column shows four trays that were classified as a ‘visit’ having occurred, with footprints and small depressions visible in the sand. The right column shows four trays that were classified as a ‘digging’ having occurred, with clear signs that sand has been moved, dug up, or in some cases removed from the container entirely.
**Figure S2**. One of the foraging ‘patches’ made up of four GUD trays used in experiment 2. Trays contain 2.5 L of sand. 3/4 trays contain 30 wheat seeds, while the other tray contains 30 lentils. In treatment enclosures, one of the wheat trays has been sprayed with a 10× camouflage solution (quantity of wheat germ oil roughly equivalent to 300 wheat seeds) and another with a 50× camouflage solution (equivalent to 1500 wheat seeds).

## Data Availability

The data that support the findings of this study are available from the corresponding author upon reasonable request.
