# Supplementary material for: Nutritional missed opportunity costs: wild house mice ( Mus musculus ) consistently consume less preferred food, with implications for control
Source: Pest Manag Sci. 2025 Sep 17;82(1):742–50. doi: 10.1002/ps.70229 (PMC12713708; doi:10.1002/ps.70229)
Supplement: Supplementary file 1 — Figure S1. Plastic containers filled with sand that represented wheat ‘patches’ used in experiment 1. The left column shows four trays that were classified as a ‘visit’ having occurred, with footprints and small depressions visible in the sand. The right column shows four trays that were classified as a ‘digging’ having occurred, with clear signs that sand has been moved, dug up, or in some cases removed from the container entirely. Figure S2. One of the foraging ‘patches’ made up of four GUD trays used in experiment 2. Trays contain 2.5 L of sand. 3/4 trays contain 30 wheat seeds, while the other tray contains 30 lentils. In treatment enclosures, one of the wheat trays has been sprayed with a 10× camouflage solution (quantity of wheat germ oil roughly equivalent to 300 wheat seeds) and another with a 50× camouflage solution (equivalent to 1500 wheat seeds). [file PS-82-742-s001.docx]

**Supplementary material –** Nutritional missed opportunity costs: wild house mice (*Mus musculus*) consistently consume less preferred food, with implications for control


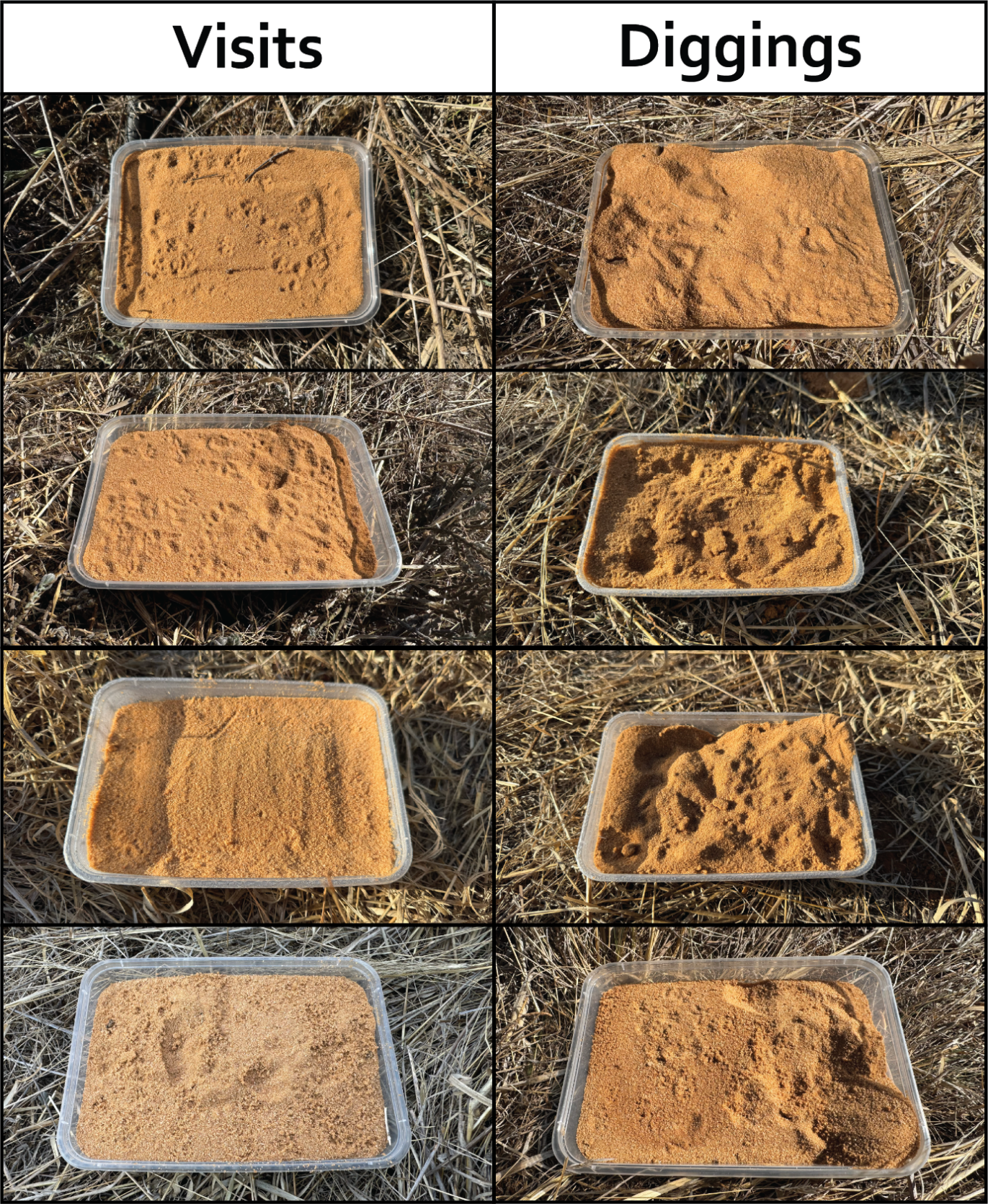


**Figure S1** Plastic containers filled with sand that represented wheat ‘patches’ used in experiment 1. The left column shows four trays that were classified as a ‘visit’ having occurred, with footprints and small depressions visible in the sand. The right column shows four trays that were classified as a ’digging’ having occurred, with clear signs that sand has been moved, dug up, or in some cases removed from the container entirely.


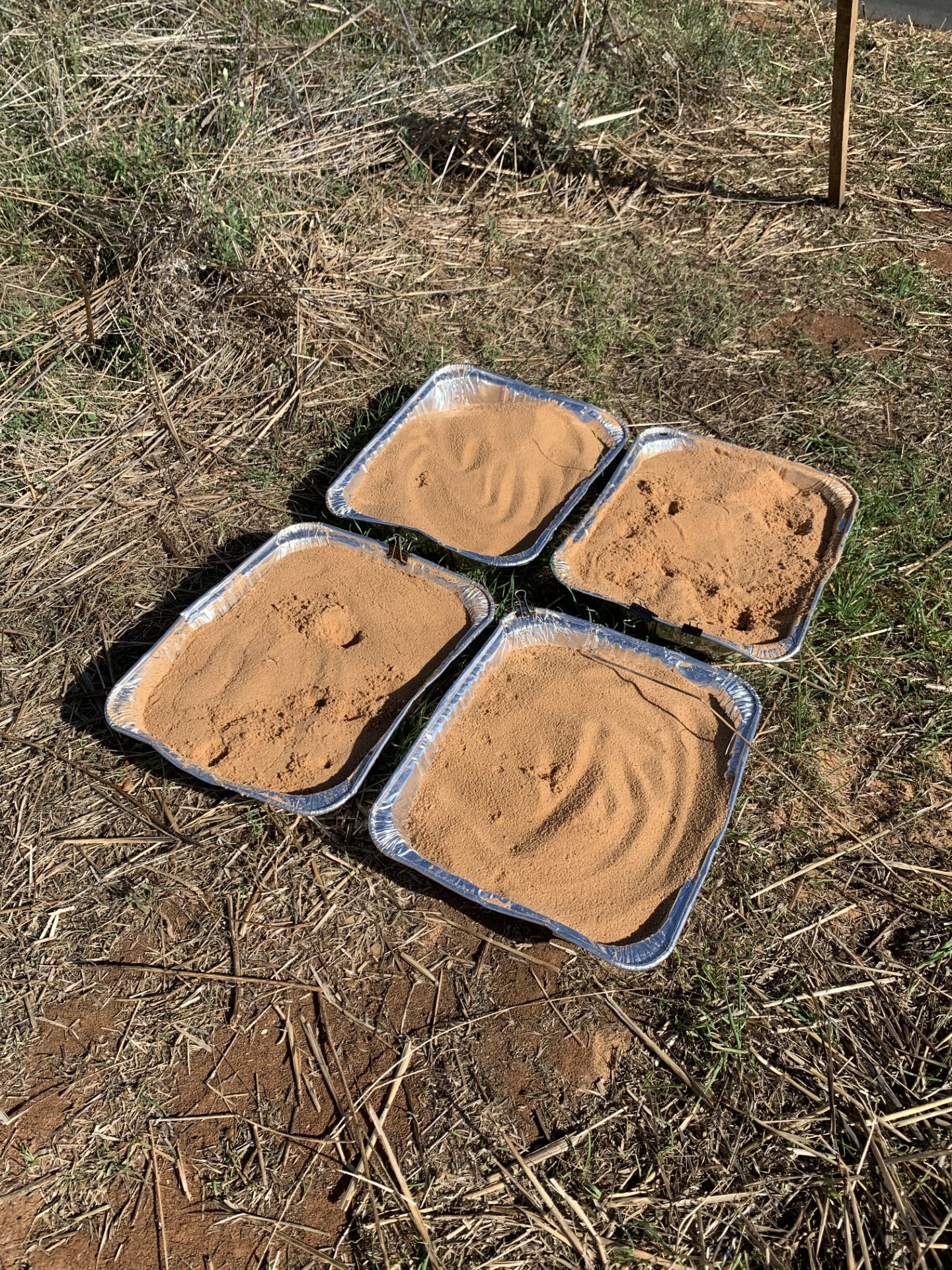


**Figure S2** One of the foraging ‘patches’ made up of four GUD trays used in experiment 2. Trays contain 2.5 L of sand. 3/4 trays contain 30 wheat seeds, while the other tray contains 30 lentils. In treatment enclosures, one of the wheat trays has been sprayed with a 10× camouflage solution (quantity of wheat germ oil roughly equivalent to 300 wheat seeds) and another with a 50× camouflage solution (equivalent to 1500 wheat seeds).
